# Social services utilisation and referrals after seeking help from health services for self-harm: a systematic review and narrative synthesis

**DOI:** 10.1136/bmjph-2023-000559

**Published:** 2023-12-07

**Authors:** Sarah Steeg, Faraz Mughal, Nav Kapur, Shamini Gnani, Catherine Robinson

**Affiliations:** 1 Centre for Mental Health and Safety, Division of Psychology and Mental Health, University of Manchester, Manchester, UK; 2 NIHR School for Primary Care Research, University of Manchester, Manchester, UK; 3 Manchester Academic Health Science Centre, University of Manchester, Manchester, UK; 4 School of Medicine, Keele University, Keele, UK; 5 NIHR Greater Manchester Patient Safety Research Collaboration, School of Health Sciences, University of Manchester, Manchester, UK; 6 Mersey Care NHS Foundation Trust, Liverpool, UK; 7 Department of Primary Care and Public Health, Imperial College London, London, UK; 8 Social Care and Society, School of Health Sciences, University of Manchester, Manchester, UK

## Abstract

**Objectives:**

Suicide prevention is a major public health challenge. Appropriate aftercare for self-harm is vital due to increased risks of suicide following self-harm. Many antecedents to self-harm involve social factors and there is strong rationale for social services-based self-harm aftercare. We aimed to review evidence for social service utilisation and referrals among people seeking help following self-harm.

**Design:**

Systematic review with narrative synthesis.

**Data sources:**

PubMed, PsycINFO, AMED, Social Policy and Practice, EMBASE, Medline, Web of Science, Social Care Online, citation lists of included articles and grey literature.

**Eligibility criteria:**

Studies of people of any age in contact with health services following self-harm, with study outcomes including referrals to or utilisation of social workers and social services.

**Data extraction and synthesis:**

Information was extracted from each included study using a proforma and quality was critically assessed by two reviewers. Narrative synthesis was used to review the evidence.

**Results:**

From a total of 3414 studies retrieved, 10 reports of 7 studies were included. Study quality was generally high to moderate. All studies were based in emergency departments (EDs) and most were UK based. In studies based solely on ED data, low proportions were referred to social services (in most studies, 1%–4%, though it was up to 44% when social workers were involved in ED assessments). In one study using linked data, 15% (62/427) were referred to social services and 21% (466/2,205) attended social services over the subsequent 3-year period.

**Conclusions:**

Overall, few patients were referred to social services after self-harm. Higher reported referral rates may reflect greater service availability, involvement of social workers in psychosocial assessments or better capture of referral activity. Social services-based and integrated approaches for self-harm aftercare are important future directions for suicide prevention. Improved links between social services and health services for people seeking support after self-harm are recommended.

## Background

Effective and prompt follow-up care for people who have self-harmed is a key component of suicide prevention strategies.^1–3^ Self-harm is a strong risk factor for suicide.^4^ Self-harm presentations to health services, therefore, present important opportunities for services to coordinate appropriate aftercare. Current evidence for effectiveness of psychosocial interventions is limited^2 5^ and there are few studies of social services-based interventions. There is consensus that self-harm management should be cross-disciplinary in nature because many people who have harmed themselves have significant personal, social and economic problems as well as mental health conditions.^2 3 6^ The nature of social services contact following self-harm is potentially wide-ranging. For example, following self-harm, adults may be assessed as requiring social work support for disability or old age, there may be child protection and welfare needs in the family or substance misuse may be evident. Given the limited evidence base for healthcare-based interventions and the significant social needs in this population, there is a strong rationale for understanding the role of social services in self-harm aftercare.

Social workers have a significant role in suicide prevention. A study examining costs of self-harm found that social care resource use accounted for 13% of all health and social care costs for people who presented to health services following self-harm.^7^ Another study found that among 174 people aged under 25 who had died by suicide, 22 had seen a social worker within mental health services.^8^ However, the specific contribution of social services in the care of people who have harmed themselves is unknown and research is limited.^9–11^ Specifically, the rates of social services used in subsequent care among people who have harmed themselves is not well understood.

People seeking help for self-harm often do so from primary care and emergency departments (EDs); therefore, these are key settings for arranging follow-up treatment.^12^ Social services often have formal involvement in the hospital management of self-harm,^13^ for example, social workers frequently conduct psychosocial assessments and arrange follow-up care for people presenting to hospital after self-harm.^14 15^ For some people seeking help, addressing social care needs is required in parallel with addressing psychological needs. Recent guidance from the National Institute for Health and Care Excellence recommends a shared approach between social care agencies and healthcare professionals when caring for people after self-harm.^2^ This approach can help ensure continuity of care for people already in contact with social services as well as contribute to a holistic consideration of people’s needs. Self-harm is linked to social and interpersonal problems as well as mental disorder,^16^ and people presenting to ED for self-harm report multiple social problems including relationship problems, drug and alcohol misuse, physical ill health, housing and employment problems and domestic abuse.^17^ Despite this, evidence regarding specific social services utilisation among people who have harmed themselves is sparse. In one study, while the sample was small, drug dependency was found to be associated with people having social worker contact and reporting suicide attempts.^11^ An association between being in the local authority care system and suicide attempt has also been reported in Sweden.^18^

In this study, we aimed to systematically identify, assess and synthesise the evidence for social service utilisation and referrals among people seeking help from health services following self-harm.

## Search Strategy And Selection Criteria

A systematic review of peer-reviewed academic research and grey literature was undertaken, with study inclusion based on the following criteria:

► Studies of people of any age in contact with health services following an episode of self-harm, including intentional self-poisoning or self-injury with or without suicidal intent. We were interested in any health service contact, including both primary and secondary care.
► Study outcomes include referrals to or utilisation of social workers and social services. This definition could include social workers based in health services and services provided by local authorities, including support from, for example, social workers, occupational therapists and support with housing and social welfare benefits.

This review was conducted and reported adhering to the Preferred Reporting Items for Systematic Reviews and Meta-Analyses (PRISMA) guidance, following PRISMA reporting items.^19^ The study protocol was registered prospectively on PROSPERO: https://www.crd.york.ac.uk/prospero/display_record.php?ID=CRD42023310285. An amendment was made to the outcomes in the registered protocol. The original planned study outcomes were ‘social care service utilisation and social care needs among people who have harmed themselves’. Several tens of thousands of articles were returned after conducting searches for these outcomes. Filtering by search terms showed that the ‘social care needs’ outcome generated a large number of returns, covering a broad set of topics. Following discussions with the author team and public contributors it was agreed that it would not be feasible to review such a large number of articles. Therefore, this review focuses on social care services utilisation; the second outcome will be assessed in a subsequent study. While we originally intended to review service utilisation among people aged 16 years and over, the included studies did not provide results for this specific age group.

### Search strategy

**Figure 1:**
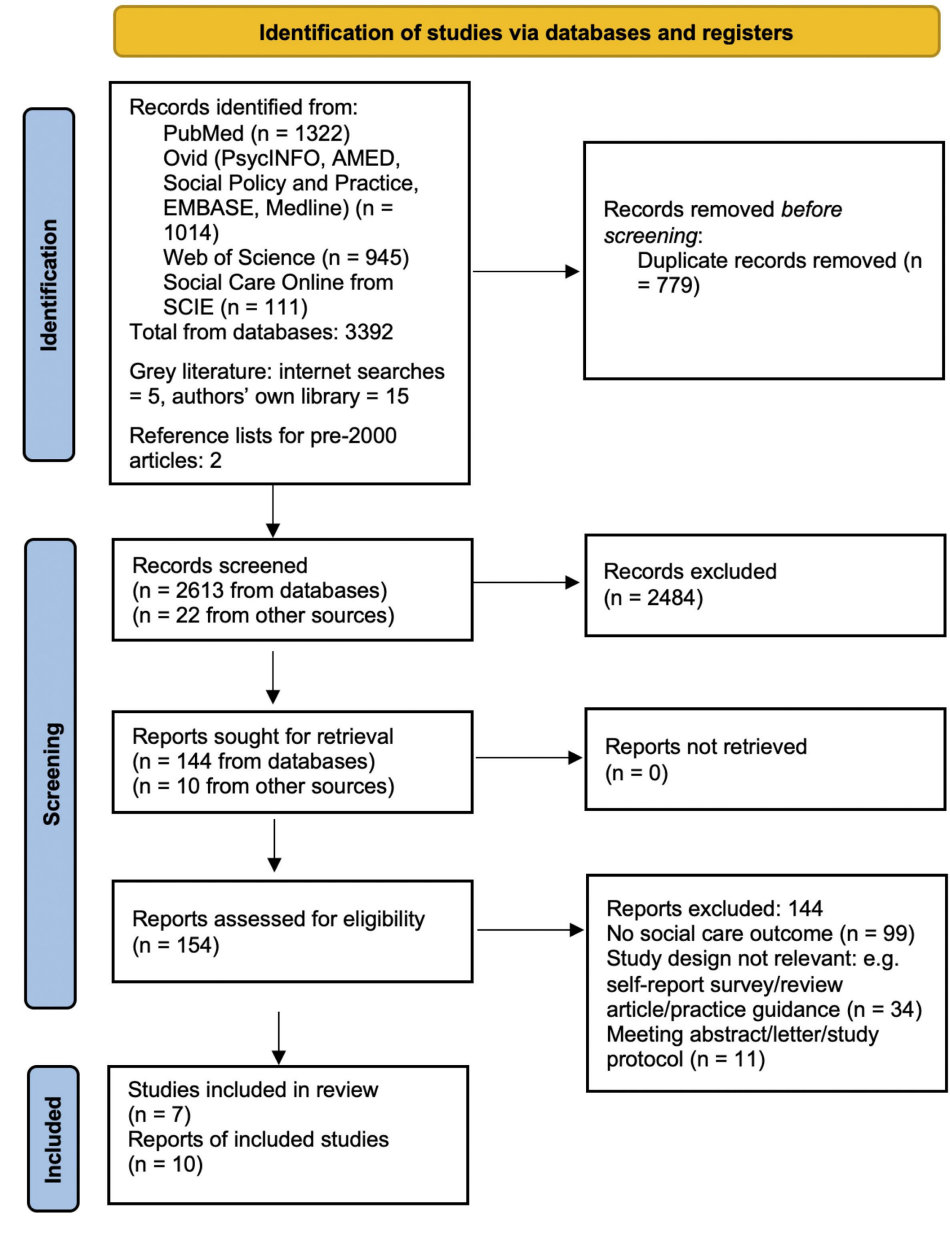
Preferred Reporting Items for Systematic Reviews and Meta-Analyses (PRISMA) flow diagram. From Page *et al*.^45^

PubMed, PsycINFO, AMED, Social Policy and Practice, EMBASE, Medline, Web of Science and Social Care Online (from SCIE databases) were searched for articles published between 1 January 2000 and end of February 2023 (figure 1). Searches were conducted in Febuary 2023. Separate searches were conducted for MeSH terms and titles/abstracts (see online supplemental file 1 for full lists of search terms). Examples of search terms included: ‘self-harm’, ‘suicidal’, ‘self-injur*’, ‘self-poison*’, ‘self-cut*’, ‘parasuicid*’ and ‘overdos*’ to capture studies reporting self-harm. The automated searches were limited to years 2000 onwards for pragmatic reasons, as recommended when several thousand returns are generated.^20^ No language restrcitions were imposed. Grey literature was searched by Google search engine and the authors’ hard copies of reports were checked. Citation lists of included articles and narrower search terms were used to search for relevant evidence published before 2000.

### Inter-rater agreement, data extraction and synthesis

Information relating to healthcare setting, patient group, study design and study findings (proportion referred to/in contact with social services) was extracted from the included studies. Critical appraisal tools (selected according to study design) from the Joanna Briggs Institute collection were used to assess the quality and risks of bias of each study.

SS screened all of the 2625 titles and abstracts and a second reviewer (FM) independently screened 10% (261). Differences in screening outcomes between the two reviewers were examined using percentage agreement and Cohen’s kappa to measure inter-rater agreement. Independent reviewing of 261 abstracts by FM led to three records recommended for retrieval (all also recommended by SS, with SS recommending an additional three records). The kappa score for these ratings was 0.66 and the level of agreement was 98.9% (258/261). Two reviewers (SS and FM) independently conducted quality assessments of all included studies. Following independent assessment, SS and FM discussed overall conclusions of quality, after which the assessment of one study was downgraded from high moderate to moderate.

Narrative synthesis was conducted guided by the Popay *et al*’s framework.^21^ This approach was used as we were interested in understanding the nature of social services utilisation after self-harm and exploring the health settings in which the evidence related to. In addition, pooling of results and meta-analysis would be unsuitable for this research question due to heterogeneity in study periods and study designs. Initially, we examined how the included studies might be organised to best describe patterns in their findings. Findings between studies, including across types of study designs, patient populations and outcome measures were then explored to analyse how these factors might influence study findings. The robustness of the evidence was then assessed by considering the strength of the overall evidence and in which populations and contexts in which it was representative.

### Patient and public involvement

Four service users and carers with lived experience of health services for self-harm worked with the research team to design the study and interpret the findings. Specifically, the public contributors worked on the study inclusion criteria and the conceptualisation of social services care following self-harm. The group (named Mutual Support for Mental Health-Research, MS4MH-R) is linked with the National Institute for Health and Care Research Greater Manchester Patient Safety Research Collaboration.

## Results

As a result of the electronic database searches, 3392 articles were identified and 2613 were identified for screening (figure 1). A total of 144 of these were assessed for eligibility resulting in four articles^22–25^ meeting the inclusion criteria and included in the review (figure 1). Two further articles published before 2000 were included following hand searches of citation lists and the adjusted (narrower) search strategy for pre-2000 evidence.^26 27^ Searches for grey literature produced 22 relevant reports, of which 10 were retrieved and four (all reported on different time periods within a single study) were selected for inclusion.^28–31^ A total of 10 reports of 7 studies were finally included in this review.

### Description of included studies

All seven studies were based in EDs and one also included a specialist self-harm unit.^24^ Five out of the seven included studies were conducted solely in the UK, one study was based in an Irish ED,^26^ and one study was based in two sites; in Oxford, UK and in Newcastle, Australia^22^ (table 1). Six studies derived the study outcome solely from information recorded in patient assessments or hospital records. One study^24^ used data from other health and social care agencies as well as the patient assessments in ED. Five studies included presentations of intentional self-harm involving self-poisoning and self-injury regardless of intent, one included self-poisoning episodes only^22^ and another included ‘suicidal behaviour’.^26^

### Findings of included studies

In five out of six of the studies using ED patient data solely, between 1% and 4% of self-harm presentations were referred to social care services. Two of these studies reported the proportion referred among those receiving a psychosocial assessment and both found referral rates of around 4%. In the study comparing management between patients assessed by psychiatric staff and those assessed jointly by psychiatric staff and social workers, the proportions were 18% (9/50) and 44% (22/50), respectively.^27^ In a study using ED data with linked data from other health and social care agencies, 15% (62/427) of people attending a self-harm unit were referred to social services and 21% (466/2205) of all hospital self-harm patients were recorded as attending social services over a 3-year period.^24^

The years the studies were conducted ranged from 1983 to 2011. There was no clear evidence for a relationship between study findings and year of study. The highest proportion of patients referred to social care services was reported in Bateson *et al*’s study using data from 1983 and 1984, which may reflect availability of services at that time or the involvement of social workers in self-harm psychosocial assessments in that study.^27^ The quality of studies was generally high to moderate (table 1). One study was assessed as low to moderate due to poor definition of the exposure ‘suicidal behaviour’ and limited details regarding data extraction.^26^

## Discussion

### Main findings

All studies included in this review were based in EDs and were mainly UK based. The quality of studies was generally high to moderate. All but one study derived the outcome data solely from information recorded in patient assessments or hospital records. Rates of referrals were generally relatively low—around 1%–4%. When actual service use data were captured, around one in five used social care services following self-harm—though evidence of this was limited to a single study. We found some evidence that when social workers were involved in conducting a psychosocial assessment, social services referral rates were higher.

### Implications and comparison with existing evidence

A systematic review of resource utilisation in the year following a hospital presentation for self-harm found that social services costs comprised 13% of the total health and social services costs,^32^ the second highest cost after inpatient psychiatry. While the relatively low proportions referred to social services found in our review may appear discrepant with Sinclair *et al*’s findings, there are several factors to consider. Most of the studies in our review relied on routinely recorded clinical data from a single service, which may have underestimated the use of social care services following self-harm. When linked data from various health and social care agencies were considered,^24^ the proportion of people in contact with social services was considerably higher, suggesting people who have harmed themselves were already in contact with social services or subsequently began receiving care. In addition, most of the studies in our review were based on all patients identified as attending the ED for self-harm, regardless of whether or not they received a psychosocial assessment. Without a psychosocial assessment, there is unlikely to be an opportunity to arrange appropriate follow-up care. Furthermore, the true needs of patients are likely to be underestimated in these studies. Previous research in 31 hospitals in England found that the proportion of patients receiving a psychosocial assessment following self-harm varied widely, from 22% to 88%.^13^ Recent evidence suggests there are multiple significant barriers to psychosocial assessment faced by people who have harmed themselves.^33^ It is possible that the low referral rates found in our review reflect greater barriers faced by people who have social services needs.

Rates of referrals to social services are likely to be influenced by the professional background of the assessor. A previous study found that the professional background of clinicians conducting self-harm assessments influenced patients’ subsequent clinical management.^34^ The presence of multidisciplinary teams, including social workers, was thought to improve quality of aftercare for people presenting to ED following self-harm.^35^ In one study included in this review, assessments conducted jointly with psychiatrists and social workers had higher rates of referrals to social services.^27^ A study conducted in the 1970s found that social workers conducting assessments following self-harm placed greater emphasis on relationship and family problems and were more likely to identify physical illness compared with junior doctors.^36^ However, more recent studies indicate that referrals for follow-up care do not always lead to offers of care after being treated in hospital for self-harm.^37^ For example, significant clinician and patient barriers to the recommended psychological therapies following self-harm have been reported.^35 38^

There was an absence of studies from primary care settings in our study. A recent review found that there was limited information relating to social services needs, and social needs more broadly, recorded in UK primary care data; this gap may partly explain why no primary care-based were identified in our review. There are recognised gaps in self-harm clinical guidelines and training for general practitioners (GPs)^39 40^ and research into social care needs and referrals to social services among patients seeking help for self-harm from their GP is needed.

It is also important to understand how referrals made to social services following ED-presenting self-harm relate to future patient outcomes such as further health and social care services use and risks of further self-harm and death by suicide and other external causes. Currently, evidence relating to such outcomes is limited. One challenge is that observational evidence regarding outcomes among people referred to social services is subject to strong limitations of confounding. For example, a study of individuals in Sweden who had received welfare interventions during childhood, such as foster care, was at higher risk of suicide attempt in adulthood, even after adjusting for important measured confounders.^18^ Reviews of social work approaches to suicide prevention have found an absence of high-quality intervention research and advocate for more qualitative evidence to guide the development of interventions.^41 42^ In one of the few studies addressing this gap, Petrakis and Joubert evaluated a social work intervention comprising assertive brief psychotherapeutic intervention alongside support linking to community services, with individuals presenting to an ED after suicide attempt.^43^ While this was not a controlled study and there was no comparison group, individuals receiving the intervention reported improvements in several domains including work, finance, relationships and living circumstances after 3 months.

Given the lack of robust evidence for healthcare services-based psychosocial interventions following self-harm, integrated approaches involving social services are an important future direction for suicide prevention. Few interventions for self-harm have involved social services, though some social work-based and integrated interventions have been associated with improvements in mental health and social circumstances.^43^ A service for men with suicidal feelings addressed financial, housing and employment problems alongside providing emotional support.^44^ The service was associated with reductions in suicidal ideation and was valued by service users.

### Strengths and limitations

This is the first review of social service referral and utilisation among people seeking help from health services following self-harm. The systematic review methodology with narrative synthesis enabled us to explore factors potentially influencing the findings reported in each study. Our research question aimed to confirm current practice and identify variation in practice, and we judged systematic review with narrative synthesis to be the most appropriate approach. However, we acknowledge that there is some overlap in the aims of systematic review and scoping review methodology, and that alternative approaches may have also been appropriate.^19^ All but one study examined referrals to social services only, with one^24^ also measuring utilisation of social services up to 3 years after a self-harm episode. While we are unable to draw conclusions based on one study, it is possible that examining referrals following a healthcare presentation for self-harm underestimates the level of social services utilisation among this patient group. The findings should be interpreted in the context of the small number of studies included in the review. In addition, the studies in this review spanned a range of time periods from 1983 to 2011 and no studies included years past 2011.

Emergency healthcare and social services in the UK faced numerous changes during and since that period. For example, in 2010/2011, a greater number of English EDs had formal arrangements with social services to provide assessments for self-harm patients than in 2001/2001.^13^ In addition, the introduction of integrated care systems (ICSs) in England from 2022 is aimed at linking National Health Service (NHS), local authority and community organisations to deliver health and care services. One of the aims of ICSs is to improve access to health and care services. It is possible that such partnerships will affect patterns of referrals following self-harm. Therefore, the findings cannot necessarily be generalised to the entire period of study nor to more recent years. The majority of studies were conducted in the UK, with one each in Ireland and Australia. The findings are unlikely to reflect international practice due to variations in service provision and the availability of health and social care services. We defined social services as care provided by social workers situated in health services, or social services provided by local authorities. However, studies generally did not define this study outcome in detail, so it was not possible to understand exactly what service people were referred to. We did not include studies of people seeking help for suicidal ideation; future research should investigate the clinical management of people presenting to services with suicidal thoughts. Finally, there was no evidence from primary care settings.

## Conclusions

In general, few patients are referred to social services after an episode of self-harm. Referral rates may be higher in instances where social workers are involved in psychosocial assessments. Involving social workers in self-harm assessments could improve links between social services and people requiring support after self-harm. Studies using data linkage to capture referral activity are likely to have greater accuracy in identifying patients referred to social services. Rates of contact with social services in the years following self-harm are likely to be higher than referral rates, and are considerably higher than in the general population, though more evidence is needed. Evidence from primary care settings is also urgently needed. There is a lack of robust evidence for healthcare services-based interventions and social services-based and integrated aftercare and interventions for self-harm are important future directions for suicide prevention; suicide prevention approaches must address societal-level factors. Future research should investigate how social care needs and social care services utilisation relate to future risks of self-harm and premature mortality in people presenting to health services.

## Supplementary Material

Data supplement

## Figures and Tables

**Table 1:** Characteristics and findings of included studies (n=7)

Study ID	Healthcare setting	Patient group	Study design	% Proportion referred to/ attended social care services	Additional information	Quality of evidence
Hiles *et al*, 2015^22^	One ED in Oxford, UK and one ED in Newcastle, Australia	Patients aged 10 years and over presenting with deliberate self-poisoning between 1997 and 2006	Cross-sectional, observational	3.8% (249/6534) referred	Study compared management in Oxford, UK and Newcastle, Australia.	High/moderate
Horrocks *et al*, 2003^23^	Two EDs in Leeds, UK	All episodes of self-harm (self-injury and self-poisoning) between first March 2000 and 31 August 2001 by people aged 12 years and over receiving a psychosocial assessment and whose follow-up arrangements were known	Cross-sectional, observational	4.1% (45/1316) referred (among assessed)		High/moderate
Keene, 2005^24^	One ED and one specialist self-harm unit in the UK	People attending the ED or specialist self-harm unit following self-harm (self-injury and self-poisoning) over a 3-year period (years not specified).	Cross-sectional, observational	15% (62/427) of people attending a self-harm unit were referred to social services 21% (466/2205) of all hospital self-harm patients were recorded as attending social services over a 3-year period	This was the only study using linked data across health and social care services. Therefore, service contact ascertainment is likely to be better captured. Both referrals and service use were included as study outcomes. Percentage of those using social services is about seven times greater among people attending hospital for self-harm than the general population.	Moderate
Gunnell *et al*, 2013^25^	31 EDs in England, UK	Patients aged 18 years and over presenting with self-harm (self-injury and self-poisoning) over a 3-month period between 2010 and 2011	Cohort, observational	2.3% (128/5624) referred (Among assessed: 3.8%, 128/3574)	Multicentre study—variation by hospital not reported.	High/moderate
Webb *et al*, 1993^26^	One ED in Dublin, Ireland	ED referrals to the department of psychiatry following suicidal behaviour during a 6-month period in 1991	Cross-sectional, observational	2.4% 9 (4/165) referred	‘Suicidal behaviour’ was not defined; it is not clear how suicidal intent was established and if self-harm with no apparent suicidal intent was included.	Low/moderate
Bateson *et al*, 1989^27^	One ED in England, UK	Patients referred to the liaison psychiatry service from the ED after self-harm (with or without suicidal intent) between 1983 and 1984	Before-and-after, controlled study (Before=liaison psychiatry service, after=joint psychiatrist-social worker service)	44% (22/50) among those assessed by psychiatric staff and social workers jointly 18% (9/50) in the sample assessed by psychiatric staff only	Among those with social workers involved in assessment, higher rates of follow-up care offered and patients were more likely to think the care had a positive impact for them.	Moderate
Bickley *et al*, 2013;^28^ Dickson *et al*, 2009;^30^ Dickson *et al*, 2011;^31^ Murphy *et al*, 2007^29^	Three EDs in Manchester, UK	Self-harm episodes presenting to ED between 2003 and 2011	Cohort, observational	2003–2005: 1.7% (18/1078) referred 2005–2007: 1.9% (52/2772) 2098–2009: 0.9% (19/2183) 2010–2011: 2% (57/2828)	Not peer-reviewed. Few details on methods so some aspects of study design unclear.	Moderate

ED, emergency department.

## Data Availability

Data sharing not applicable as no datasets generated and/or analysed for this study. No data are available. The search terms used in each of the databases are provided in online supplemental file 1.

## What Is Already Known On This Topic

⟹ Appropriate aftercare for self-harm is key to addressing the major public health challenge of suicide prevention. However, evidence for healthcare-based approaches is limited. Addressing social problems experienced by people who have self-harmed is likely to be an important feature of aftercare but little is known about social services contact following self-harm.

## What This Study Adds

⟹ This narrative systematic review found that few patients were referred to social services after self-harm. Availability of services, involvement of social workers in psychosocial assessments and access to data capturing referral activity are all likely to influence reported referral rates.

## How This Study Might Affect Research, Practice Or Policy

⟹ Social services-based and integrated approaches for self-harm aftercare are important future directions for suicide prevention. Involving social workers in self-harm assessments could improve links between social services and people requiring support after self-harm.
