# Supplementary material for: Social services utilisation and referrals after seeking help from health services for self-harm: a systematic review and narrative synthesis
Source: BMJ Public Health. Author manuscript; Available in PMC 2024 Jan 18. (PMC7615544; doi:10.1136/bmjph-2023-000559)
Supplement: Data supplement [file EMS192778-supplement-Data_supplement.pdf]

Supplement 1: Search strategies

Database	Search terms
PubMed	1 = (((self-harm[MeSH Terms]) OR (Self-Injur*[MeSH Terms]) OR (Self Harm[MeSH Terms]) OR (Self injur*[MeSH Terms]) OR (suicid*[MeSH Terms])) AND ((Delivery of Health Care[MeSH Terms]) OR (Health Services Research[MeSH Terms])) AND (Social Care[MeSH Terms]))) OR (((self-harm[MeSH Terms]) OR (Self-Injur*[MeSH Terms]) OR (Self Harm[MeSH Terms]) OR (Self injur*[MeSH Terms]) OR (suicid*[MeSH Terms])) AND (Health Services[MeSH Terms]) AND ((Social Work[MeSH Terms]) OR (Social Welfare[MeSH Terms]) OR (Social Care[MeSH Terms])))<br><br>2 = (((self-harm[Title]) OR (Self-Injur*[Title]) OR (self-poison*[Title]) OR (self-cut*[Title]) OR (parasuicid*[Title]) OR (overdos*[Title]) OR (suicidal[Title]))) AND ((("residential care"[Title/Abstract]) OR (carer[Title/Abstract]) OR ("social care"[Title/Abstract]) OR ("social services"[Title/Abstract]) OR ("social work*"[Title/Abstract]) OR (reablement[Title/Abstract]) OR ("personal care"[Title/Abstract]) OR (safeguard*[Title/Abstract]) OR (welfare[Title/Abstract]) OR (refer*[Title/Abstract]) OR ("clinical management"[Title/Abstract]))) AND ((("Emergency department"[Title/Abstract]) OR ("Emergency room"[Title/Abstract]) OR (hospital*[Title/Abstract]) OR ("secondary care"[Title/Abstract])) OR ("general practice"[Title/Abstract]) OR ("primary care"[Title/Abstract])))<br><br>1 OR 2
Web of Science	(TI=(self-harm) or TI=(suicidal) or TI=(self-injur*) or TI=(self-poison*) or TI=(self-cut*) or TI=(parasuicid*) or TI=(overdos*)) AND ((TS=(refer*) OR TS=("clinical management")) OR (TS=("social care") OR TS=("social work") OR TS=(social service*))) AND (TS=(Emergency department) OR TS=(emergency room) OR TS=(hospital*) OR TS=("secondary care") OR TS=(general practic*) OR TS=("primary care"))
Ovid	((self-harm or suicidal or self-injur* or self-poison* or self-cut* or parasuicid* or overdos*).ti,kw. and (residential care or reablement or "personal care" or welfare or refer* or "clinical management" or safeguard* or "social care" or "social work*" or social service*).ti,ab,kw. and (Emergency department or emergency room or hospital* or "secondary care" or general practic* or primary care or "health service*").ti,ab,kw.) or ((self-harm or suicidal or self-injur* or self-poison* or self-cut* or parasuicid* or overdos*).ti,kw. and ((refer* or "clinical management").ti,ab,kw. or ("social care" or "social work*" or social service*).ti,kw.) and (Emergency department or emergency room or hospital* or "secondary care" or general practic* or primary care).ti,ab,kw.)
